# Crop: The Black Box of Mosquito Vector Fitness

**DOI:** 10.3390/insects17030234

**Published:** 2026-02-25

**Authors:** Ainhoa Rodriguez-Pereira, Frances M. Hawkes, S. Noushin Emami

**Affiliations:** 1 Natural Resources Institute (NRI), University of Greenwich, Gillingham ME4 4TB, UK; f.m.hawkes@greenwich.ac.uk; 2 Department of Vector Biology, Liverpool School of Tropical Medicine (LSTM), Liverpool L3 5QA, UK; 3 Molecular Attraction AB, 17154 Stockholm, Sweden; 4 Department of Microbiology, Tumor and Cell Biology (MTC), Karolinska Institute, 17165 Stockholm, Sweden

**Keywords:** crop, mosquito, digestion, immunity, microbiome, vector control

## Abstract

In mosquitoes, digestion involves the foregut (including the crop), midgut, and hindgut, with the midgut and crop playing important roles in processing sugar and blood meals. The well-studied midgut is a known major pathogen entry point; however, the less-explored crop may affect vector fitness and immunity. This review provides an overview of the anatomy and function of the crop, before drawing together the current state of knowledge of (I) the crop’s role in digestion, (II) its immune function, and (III) the importance of the crop microbiome and its potential role in mosquito fitness. After decades of chemical control, vector management must move beyond immediate disease prevention toward a global approach that considers mosquito biology and the crop’s diverse roles. This may make it a suitable target for new innovations by providing insights into detoxification mechanisms, microbiome-mediated functions, and their potential combined effects on vectorial capacity. Future research is needed to better understand crop function.

## Simple Summary

With the growing challenges posed by mosquito-borne diseases, a deeper understanding of mosquito physiology is essential for developing innovative strategies to control both vectors and the pathogens they transmit. This review assesses current insights and pertinent gaps in our understanding of the mosquito crop, informed by mosquito research and comparisons with analogous organs in other insects. It highlights the crop’s roles in digestion, immunity, and microbiome-driven fitness effects, underscoring its status as an overlooked yet potentially influential organ for future study.

## 1. Introduction

Mosquitoes are considered the deadliest animal on Earth due to their role in transmitting pathogens between mosquitoes and humans [1]. Vector-borne diseases account for more than 17% of all infectious diseases and cause more than 700,000 deaths annually [2]. Interventions that reduce mosquito populations and/or limit transmission are the cornerstones of mosquito-borne disease control. Core interventions include chemical vector control, such as insecticide-treated nets, and indoor residual spraying, larval source management (LSM) and repellents [3], as well as genetic and symbiont-based approaches. However, the increase in insecticide resistance and changes in the biting and host-seeking behaviour of mosquito populations have highlighted new challenges in the field, including associated costs, potential environmental issues, and reduced effectiveness [4,5]. More diverse approaches are required for controlling vectors. A recently developed strategy that is already being rolled out involves the use of the bacterial endosymbiont *Wolbachia*. This bacterium is present in insects and has been found to reduce vector competence in *Aedes* mosquitoes, thereby reducing arbovirus transmission [6]. Variable effects on arbovirus, including members of the genus *Alphavirus*, have been observed [7]. Attractive Targeted Sugar Baits are also under development. They exploit the natural nectar-feeding behaviour of mosquitoes to deliver mosquito-specific toxins, killing the insects and thereby reducing their vectorial capacity [8]. This strategy targets both female and male mosquitoes, as nectar drinking is not a sex-specific behaviour [9]. The use of transgenic mosquitoes is another strategy currently being trialled to suppress/replace wild vector populations with new populations carrying traits that reduce or eliminate transmission [10], but this strategy often faces challenges, including regulatory hurdles, ecological concerns, and community acceptance.

A deeper understanding of mosquito vector–pathogen interactions is key to identifying new strategies for vector and pathogen control. Much of the knowledge of mosquito physiology and vector–pathogen interactions focuses on the blood-feeding physiological response, driven by the peripheral nervous system and the midgut, which serves as the initial destination of blood meals upon ingestion. Dengue virus (DENV), Zika virus (ZIKV), and West Nile virus (WNV) infect midgut epithelial cells, where they replicate before being released into the haemocoel and migrating to other tissues [11]. In *Plasmodium*, the malaria pathogen, the sporogonic cycle is a complex battle for invasion of the mosquito midgut. Gametocytes ingested in a blood meal form gametes and ookinetes, but only a few succeed in infecting the mosquito midgut, creating a bottleneck. Then developing into oocysts, where massive replication occurs, sharply increasing parasite numbers before they cross the midgut wall [12,13]. Mosquito digestion, however, does not exclusively involve the midgut. The crop, or ventral diverticulum, is an organ in the insect digestive system that initiates the digestion of non-blood meals in female and male mosquitoes but is somewhat overlooked in mosquito digestion research. This organ can be considered a ‘black box’ in mosquito infection and digestive physiology, describing a system with a clear ‘input’, the sugar meal, but with limited mechanistic insight into how it might alter ‘outputs’ such as mosquito fitness, from digestion to immunity, as well as crop microbiome-mediated processes. Crucial roles are beginning to emerge in recent literature. This review aims to explore some of the exciting directions in which mosquito crop research could be leading, both in terms of the fight against mosquito-borne diseases and in deepening our understanding of insect digestive physiology, particularly (I) the role of the crop in digestion, (II) the role of the crop in mosquito immunity (III) and the roles of the crop microbiome.

## 2. Roles of the Crop: Mosquito Dual-Feeding

### 2.1. What Is the Crop?

The mosquito’s digestive system is divided into foregut, midgut, and hindgut. The foregut includes the pharynx, oesophagus, and crop; the midgut is composed of the anterior midgut and posterior midgut. Once a nectar or other sugar meal is imbibed, it passes through the oesophagus and into the crop, where it is redistributed to the midgut for further digestion. The entire process takes approximately 72 h, with active digestion occurring approximately 12 h post-ingestion [14]. The crop is not exclusive to mosquitoes; it is a conserved anatomical organ across many insects, although its functional integration is more clearly pronounced in hematophagous insects.

**Figure 1:**
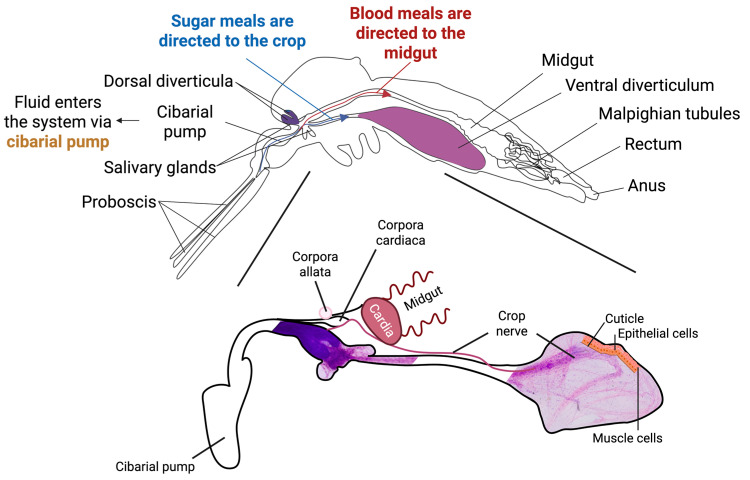
Depiction of the Mosquito Crop within the Digestive System and its Morphology and Composition. The mosquito crop (diverticulum, shown in purple) is lined by an epithelial layer beneath a chitinous cuticular intima, surrounded by muscle fibres and innervated by crop nerves. The crop contains six pumps and five sphincters [21], anatomy inferred from *Glossina*. Blood meals are directed to the midgut (red), while sugar meals are directed to the crop (blue). Fluid enters the system via the cibarial pump (yellow), which has been suggested as a key regulator of meal routing [17].

In mosquitoes, the crop stores non-blood meals, such as nectar and other sugars, for digestion. An exhaustive depiction of the crop within the mosquito digestive system is shown in Figure 1. Meals are then processed and digested by midgut-mediated absorption. The rate at which non-blood meals empty from the mosquito crop into the midgut varies with sugar concentration, with higher concentrations slowing the process [15,16]. The consensus is that liquids imbibed by the mosquito are directed to the specific organ via a switching mechanism that exists within the digestive system. This mechanism directs some meals directly to the midgut, bypassing the crop in response to specific stimuli, although the underlying mechanism remains unclear [17].

Phagostimulants such as ATP or sucrose regulate meal destination in mosquitoes, sometimes leading to misdirection of meals to the midgut or crop, respectively [18]. This phenomenon might have downstream effects on survival, fitness, or reproductive output. Further research was carried out by Bryant et al. [19], who demonstrated that the loss of microRNA-275 led to a phenotype in which the blood meal was found in both the crop and the anterior midgut, undigested, with no peritrophic matrix or food bolus. Hypothesising that the miR-275 phenotype might have a defect in muscle function, causing the regurgitation of undigested blood into the crop, thus highlighting a potential role of miRNA in crop physiology. Lastly, the addition of a K_ATP_ channel activator (pinacidil) to the blood meals of *Aedes aegypti* led the blood meals to be misdirected to the crop, instead of the midgut. Revealing the potential role of K_ATP_ channels in meal destination regulation [20].

### 2.2. Anatomy and Function

The insect crop consists of two diverticula that extend from the main crop lumen. They are called the ventral and dorsal diverticula. The dorsal diverticulum is composed of two lobes positioned before the opening of the ventral diverticulum, which is formed of a bilobed sac. The ventral diverticulum is innervated by nerve bundles originating from the nervous system, which is connected to the brain and suboesophageal ganglia. The nervous bundle is also connected with the corpora cardiaca consisting of axonal projections from brain neurosecretory cells that store and release hormones into the hemolymph and contribute to metabolic regulation [22]. This system is analogous to the brain–gut axis of vertebrate species [21,23,24]. The crop is lined by a chitin cuticle and a protein matrix, which have not yet been well described [16]. Surrounding the crop is a muscular system that regulates the flow of ingested material between the crop duct and the crop lobes, comprising approximately 6 pumps and 5 sphincters [21]. Stretch-activated channels regulate the muscles within the crop lobes [16], which open or close sphincters upon distention of the organ, to ensure the liquid is either stored in the crop or sent forward towards the midgut [16,25,26]. Some pumps within the crop are key to understanding this organ. P4, which is located at the entrance of the ventral diverticulum, is a major pump. This pump is modulated by neurotransmitters, including serotonin, which has been shown to be transported through the hemolymph in insects such as blowfly (Diptera: Calliphoridae) [25]. P2 is located in the foregut, near the opening to the midgut. P4 and P2 are the pumps that move blood directly into the midgut for digestion. Movements within the crop are controlled by hydrostatic pressure cues and mediated by peristaltic movements. Calkins et al. (2017) [16] reviewed the pathways implicated in the potential contractile properties of the crop in mosquitoes.

## 3. Role of the Crop in Digestion

Recent literature on vectorial capacity and vector–pathogen research has examined mosquito blood-feeding physiology, the primary driver of vector transmission success [27]. Blood-feeding mostly occurs in female mosquitoes to support oviposition [28]. When female mosquitoes imbibe a blood meal, the liquid is pumped from the labrum into the oesophagus and then directed into the mosquito midgut, where the peritrophic membrane is formed. This membrane surrounds the food bolus and compartmentalises nutrient absorption and digestion [29], while protecting the midgut from potential toxins or pathogens [30].

Mosquitoes also require a source of carbohydrates from food to cover the essential energetic requirements of flight, movement, mating, reproduction, and general fitness. This is the case for both sexes [31]. Mosquitoes primarily obtain carbohydrates from nectar, which is composed predominantly of sucrose in varying concentrations. Melezitose, glucose and fructose, among others, can also be present in lower concentrations [32]. Primary and secondary metabolites are also present in nectar, such as amino acids, lipids, vitamins, proteins, terpenes, alkaloids, and phenolics [33].

Mosquitoes imbibe nectar meals directly into the crop, from which the contents are selectively released into the midgut for digestion [17]. This contrasts with blood meals, which are transported directly into the midgut [34,35]. The primary role of the crop was therefore believed to be exclusively that of a storage organ for nectar meals prior to midgut digestion. However, saliva ingested with the sugar meal was shown to contain invertase, lactase, maltase, and amylase [36], indicating that these enzymes begin digestion immediately upon ingestion and that this continues within the crop before reaching the midgut [26,37].

Further research on other Diptera showed potential enzymatic secretions within the crop. Sinha (1976) [34] found galactosidase, fructosidase, prolinase and glycylglycine dipeptidase, among other enzymes, within the crop of the housefly, *Musca domestica*; these enzymes are reportedly not present in the salivary glands, and regurgitation is unlikely, and it was concluded that the source of these enzymes is the crop itself [34]. Moreover, other enzymes have been identified in the *Glossina* crop, including proteolytic enzymes [35]. In *Phlebotomus papatasi*, *Leishmania major* parasites express α-amylase and α-glucosidase in the salivary glands, crops and guts of the insect host [37]. Furthermore, chitinase, *N*-acetylglucosaminase, and cellulolytic enzymes enable the survival of *Leishmania major* in the *Phlebotomus papatasi* gut and care expressed by the parasite in the vector gut, but also aids the fly with the digestion of complex starches and cellulose [38].

However, it is unclear if the crop tissue secretes these enzymes. It is more likely that these digestive enzymes are introduced from the meal, as floral nectar can contain enzymes such as invertases, phosphatases and oxidases [39]. They can also be introduced via the meal’s microbiome. Whether the crop has additional digestive functions, and the mechanisms underlying these functions remain unconfirmed.

Similarly, possible functions of the crop cuticular lining require further elucidation. Stoffolano and Haselton (2013) [21] suggest that the crop may play a role in the secretion of the cuticular lining, a thin layer of chitin microfibrils embedded in a protein matrix that lines the crop. However, further research is needed to fully understand this secretion process. Another potential role of the crop in digestion was described by Clements (1992) [40], who reported that, within the crop, sugars partially hydrolyse into hexoses, which are further utilised in the synthesis of glycogen and fatty acids and in lipogenesis [40,41].

Advances in technology have enabled new and interesting findings in crop physiology to come to light. Hixson et al. (2022) [42] showed, via transcriptomic analysis, that the crop and hindgut of *Aedes aegypti* differentially express genes involved in chitin and lipid metabolism, ion transport and CLIP-domain serine endopeptidases. Additionally, the crop is the gut region with the most significant investment in lipid carrier proteins and fatty acid transporters [42]. This implies that the role of the crop might not exclusively be that of carbohydrate storage and digestion. Additional roles might be attributed to the crop, such as in lipid metabolism, where early digestion of trace lipids from nectar sources may be taking place.

Although it is suggested that the crop not only stores nectar meals but also starts carbohydrate digestion, the mechanism behind crop-mediated carbohydrate digestion and whether other digestive roles are carried out is not yet known. Further research should focus on the crop’s metabolic profile and on the importance of this organ in sugar digestion, the mechanisms by which salivary enzymes engage in meal digestion within the crop, and the interplay between these enzymes and the crop microbiome.

## 4. Role of the Crop in Mosquito Immune Response

The crop acts as a physical barrier that protects the insect from external predators. When a nectar meal is ingested, it does not get pumped directly into the midgut, but rather it is directed at the crop, where it gets slowly released into the midgut [16], preventing pathogens and toxins from interacting with the midgut epithelium directly.

The Midgut Infection Barrier (MIB) prevents pathogens from invading epithelial midgut cells or inhibits their replication if invasion occurs [43,44]. Although blood meals are directly relevant for pathogen transmission, sugar meals are indirectly important for vector competence. One hypothesis suggests that crop storage of sugar meals and selective exclusion of blood help limit pathogen load in the midgut by partitioning feeding types, thereby preventing midgut epithelial infection in a manner similar to the MIB [45]. This could occur through the compartmentalisation of pathogens or toxicants acquired from nectar, thus safeguarding the mosquito midgut from infection. Additionally, research has shown higher levels of Toll pathway recognition proteins in the crop compared to the midgut or other tissues in *Aedes aegypti*. This pathway is known to regulate resistance to dengue virus infection [46] and contributes to midgut barriers against *Plasmodium* spp. ookinetes [47], but is also a well-known innate immune pathway in mosquitoes, activated primarily by fungi and Gram-positive bacteria [48]. While the crop’s role as an immune barrier in mosquito innate immunity is plausible, further research is needed to determine if additional pathways are enriched in the crop to combat pathogens and infection.

Beyond its role in providing a physical barrier between incoming sugar meals and the midgut, emerging literature highlights the importance of nectar meals for mosquito immunity and vectorial capacity. Particularly, sugar feeding has been discussed as a potential modulator of vectorial capacity [27]. An example of this relationship has been observed in *Lutzomyia longipalpis,* where *Leishmania mexicana* parasites require sugar meals to meet energetic demands during development [49]. Additionally, sugar feeding enables the *Leishmania* parasite to migrate toward the stomodeal valve by providing stimulus-guided cues [50].

In mosquito, after looking at different components such as survival, biting rate and reproductive capacity, among others, it was concluded that vectorial capacity is decreased in environments with readily accessible sugar and discusses the potential contribution of nectar components in melanisation, an innate immune process in mosquitoes, with some of them potentially serving as precursors or regulators of melanogenic pathways [27]. Whether this is a direct effect of sugar feeding or an indirect effect of increased blood feeding under low-sugar-availability environments is unknown.

## 5. The Roles of the Crop Microbiome

Following the discovery of the endosymbiont *Wolbachia* as a vector-control strategy, research has focused on investigating the insect microbiome and its role in mosquito fitness and physiology. Crop microbiome research remains limited (Table 1), underscoring the need for further exploration of the potential roles of the gut microbiome in crops.

The first isolation of microorganisms from the *Aedes aegypti* crop identified Gram-positive diplococci and Gram-negative rods, including *Serratia* spp., *Bacillus* spp., *Bacillus subtilis*, and *Pichia caribbica* [54]. Another study on *Aedes albopictus* reported similar diversity across the digestive organs, crop, and midgut [58]. A more recent study further characterised the bacterial communities in the crop and midgut of *Aedes aegypti* and observed a trend for smaller yet more diverse bacterial communities in the crop, with lower variability between replicates. Crop tissues were primarily dominated by bacteria from the family *Acetobacteraceae* (acetic acid bacteria [AAB]), such as *Asaia* and *Tanticharoenia*, which can catabolize sugars and produce acidic compounds (e.g., acetic acid). AAB could influence bacterial diversity in the crop by altering its pH [55]. Seabourn et al. (2023) found niche-driven assembly of the microbiome across tissues in *Aedes albopictus*, suggesting specialist associations between environment-specific bacteria and their target tissues [61]. Supporting the role of bacterial communities’ contribution to mosquito digestion, particularly within the crop. Adding another piece to the puzzle of reconstructing crop functions within mosquito physiology and digestion.

The mosquito microbiome not only influences digestive processes. The natural and experimental microbiota of *Anopheles gambiae* can influence the insect’s susceptibility to *Plasmodium* infection, and this effect occurs during ookinete invasion. The immune response mediated by the ClipA9 gene, which regulates serine protease activity, is regulated by the microbiome [62]. In particular, bacteria of the *Enterobacter* and *Chromobacterium* species have been shown to resist *Plasmodium* spp. infection in *An. gambiae* [63,64].

Interactions between the gut microbiome and infection permissiveness and fitness, focusing on vectorial capacity reduction and disease transmission blockage, have been extensively reported [65,66,67,68,69]. The gut-associated symbiotic bacterium *Serratia marcescens* has been shown to contribute to mosquito immunity. In *Anopheles stephensi*, colonisation with the *Serratia* spp. strain Y1 induced genes associated with the Toll and Imd immune pathways, resulting in inhibition of *Plasmodium berghei* infection in the midgut [70]. Additionally, RNA-seq of sugar-fed mosquitoes has shown high expression of orthologs of the Toll pathway, as observed in the natural microbiota of *Aedes aegypti*. Has also been shown to modulate dengue virus infection, potentially via the Toll-like immune pathway [46]. Notably, *Serratia* spp. species have been reported to dominate the microbial community within the mosquito crop [55]. This evidence suggests that the bacterium’s presence in the crop may contribute to the immune response. However, the extent of this relationship remains unknown.

Some symbiotic bacteria in insect guts can play a role in degradation and resistance to xenobiotics [71,72]. Two bacteria of particular interest in this context are present in the crop: *Bacillus* spp. and *Serratia* spp. *Bacillus cereus* in the *Plutella xylostella* gut or *Serratia* spp. in the *Dendroctonus ponderosae* beetle gut can degrade xenobiotics such as acephate or terpenoids, respectively [73,74,75,76]. *Bacillus cereus* has also been linked to organophosphate (OP)- resistant *Anopheles albimanus* mosquitoes, showing an increase in OP-degrading enzymes, including carboxylesterases, among others, in the mosquito metagenome [75]. In the *Aedes aegypti* mosquito, lambda-cyhalothrin insecticide resistance has been linked to midgut bacteria [76]. *Serratia* spp. and *Bacillus* spp. are present in the crop and could play a role in xenobiotic processing within the mosquito crop; however, this link has not yet been explored. New studies should focus on the novel contributions of the gut microbiome, particularly on species that colonise the crop.

## 6. Conclusions

This review presented the known and unknown aspects of mosquito crop research, unlocking the potential of this enigmatic component of mosquito physiology. Traditionally regarded as merely a storage site, the crop may play a more dynamic role, as evidenced by the presence of carbohydrate-digesting enzymes and transcriptomic data indicating active lipid and fatty acid metabolism. Nonetheless, the crop’s specific contributions to digestion remain poorly understood.

Emerging research has highlighted the importance of nectar meals containing both carbohydrates and plant secondary metabolites that affect mosquito immune responses and may increase innate immune responses, such as melanisation. The involvement of the crop in these responses has not been demonstrated; however, as the primary organ for nectar storage, it is an interesting avenue to explore whether this organ plays a role in initiating these immune responses.

Lastly, the crop not only has a microbiome, but it also differs from the midgut microbiome. To date, no research has examined how the crop microbiome affects crop functions, nor how it might affect vectorial capacity/pathogen transmission within the mosquito. Linking these could help establish new routes for developing effective paratransgenic tools to reduce vector competence or fitness. A lack of understanding of the interplay between the gut microbiome and the crop, which functions as the primary storage organ, is a major impediment to harnessing the full potential of this organ in the development of future vector- or pathogen-control strategies. Furthermore, a deeper understanding of the crop’s intricate metabolomic profiles could yield invaluable insights into this frequently misunderstood organ.

## Figures and Tables

**Table 1:** Bacterial species identified in the mosquito crop and their functions (predicted or tested).

Family	Genus/Lineage	Mosquito Species	Potential Functions	Publications
Acetobacteraceae	*Asaia*	*Aedes aegypti*, *Anopheles stephensi*	Activation immune genes, carbon metabolism	[51,52,53]
Acetobacteraceae	*Tanticharoenia*	*Aedes aegypti*	Oxidations and fermentations of sugars and alcohols	[54,55]
Weeksellaceae	Elizabethkingia	*Aedes aegypti*	Not known	[54,55]
Bacillaceae	*Bacillus subtilis*	*Aedes aegypti*	Not known	[55]
Yersiniaceae	*Serratia*	*Aedes aegypti*	In midgut, disrupt feeding behavior, including blood-feeding propensity	[55,56]
Saccharomycetaceae	*Pichia caribbica*	*Aedes aegypti*	Not known	[55]
Bacillaceae	*Bacillus* sp.	*Aedes aegypti*	Deglycation, protein breakdown, and perhaps local pH shifts	[55,57]
Erwiniaceae	“ERWIN1” (unclassified)	*Aedes albopictus*	Not known in mosquito, nutritional and defensive roles in other insects	[58,59]
Rhizobiaceae	*Mesorhizobium* sp.	*Aedes albopictus*	Not known	[58]
Enterobacteriaceae	“ENTERO1” (unclassified)	*Aedes albopictus*	In midgut, immune influence arboviral and malaria-parasite dynamics	[58,60]
Halomonadaceae	“HALO1” (unclassified)	*Aedes albopictus*	Not known	[58]
Bradyrhizobiaceae	*Bradyrhizobium* sp.	*Aedes albopictus*	Not known	[58]
Rubrobacteraceae	Rubrobacter	*Aedes aegypti*	Not known	[55]

## Data Availability

No new data were created or analysed in this study.

## Abbreviations

The following abbreviations are used in this manuscript:

DENV	Dengue virus
ZIKV	Zika virus
WNV	West Nile virus
MIB	Midgut Infection Barrier
CDIAL	Cinnamodial
